# Heterologous Expression of a Novel *Zoysia japonica* C_2_H_2_ Zinc Finger Gene, *ZjZFN1*, Improved Salt Tolerance in Arabidopsis

**DOI:** 10.3389/fpls.2018.01159

**Published:** 2018-08-14

**Authors:** Ke Teng, Penghui Tan, Weier Guo, Yuesen Yue, Xifeng Fan, Juying Wu

**Affiliations:** ^1^Beijing Research and Development Center for Grass and Environment, Beijing Academy of Agriculture and Forestry Sciences, Beijing, China; ^2^Turfgrass Research Institute, Beijing Forestry University, Beijing, China; ^3^Department of Plant Biology, University of California, Davis, Davis, CA, United States

**Keywords:** *Zoysia japonica*, C_2_H_2_ zinc finger protein, transgenic Arabidopsis, salinity tolerance, RNA-sequencing

## Abstract

Growing evidence indicates that some grass species are more tolerant to various abiotic and biotic stresses than many crops. Zinc finger proteins play important roles in plant abiotic and biotic stresses. Although genes coding for these proteins have been cloned and identified in various plants, their function and underlying transcriptional mechanisms in the halophyte *Zoysia japonica* are barely known. In the present study, *ZjZFN1* was isolated from *Z. japonica* using RACE method. Quantitative real time PCR results revealed that the expression of *ZjZFN1* was much higher in leaf than in root and stem tissues, and induced by salt, cold or ABA treatment. The subcellular localization assay demonstrated that ZjZFN1 was localized to the nucleus. Expression of the *ZjZFN1* in *Arabidopsis thaliana* improved seed germination and enhanced plant adaption to salinity stress with improved percentage of green cotyledons and growth status under salinity stress. Physiological and transcriptional analyses suggested that ZjZFN1 might, at least in part, influence reactive oxygen species accumulation and regulate the transcription of salinity responsive genes. Furthermore, RNA-sequencing analysis of *ZjZFN1*-overexpressing plants revealed that *ZjZFN1* may serve as a transcriptional activator in the regulation of stress responsive pathways, including phenylalanine metabolism, α-linolenic acid metabolism and phenylpropanoid biosynthesis pathways. Taken together, these results provide evidence that *ZjZFN1* is a potential key player in plants’ tolerance to salt stress, and it could be a valuable gene in *Z. japonica* breeding projects.

## Introduction

Salinity stress is becoming an increasing abiotic stress that limits crop yield and plant distribution worldwide (Zhu, 2001; Deinlein et al., 2014). Growing evidence indicates that some forage grass and turfgrass species are more tolerant to various abiotic and biotic stresses than many cultivated crop varieties (Shi et al., 2013a). Recently, several salt stress responsive genes isolated from grass species were reported as active participants in environmental responses (Huang et al., 2014). By binding to distinct *cis*-regulatory elements, TFs including NAC, WRKY, MYB, bZIP, and zinc finger TFs play a key role in regulating plant responses to salinity stress (Deinlein et al., 2014).

Zinc finger proteins represent a large family of TFs in plants (Kielbowicz-Matuk, 2012). According to the number and location of the histidine and cysteine residues, which surround the zinc atom to form a “zinc-finger structure,” zinc finger proteins are classified into several subfamilies, such as C_2_H_2_, CCCH, C_2_HC, C_2_HC_5_, and C_4_ (Laity et al., 2001). Specifically, 176 and 189 C_2_H_2_ zinc finger proteins were identified in Arabidopsis and rice, respectively, supporting that C_2_H_2_ is one of the most abundant families of zinc finger proteins in plants (Huang et al., 2007; Yin et al., 2017). These C_2_H_2_ zinc finger proteins have been functionally well-characterized in model plants and crops, and reported to play key roles in plant development and responses to environmental stresses. For instance, constitutive expression of *ZAT7* or *Zat10* enhanced the tolerance of Arabidopsis to salinity (Mittler et al., 2006; Ciftci-Yilmaz et al., 2007). Overexpression of *ZFP179* improved salt tolerance but caused hypersensitivity to exogenous ABA in rice (Sun et al., 2010). Overexpression of *ZAT18* (Yin et al., 2017) and *GsZFP1* (Luo et al., 2012) enhanced drought tolerance of Arabidopsis, while *GmZFP3* negatively regulated its drought tolerance (Zhang D.Y. et al., 2016). While *AtZAT6* can modulate biotic and abiotic stress tolerance by activating the SA pathway (Shi et al., 2014), ZFP36 plays an important role in ABA induced antioxidant response in rice (Zhang et al., 2014). However, the functions of C_2_H_2_ zinc finger proteins in stress responses and the mechanism underlying the regulation of transcription in stress related genes is still unclear, especially in grasses.

Zoysiagrass (*Zoysia* spp. Willd.), one of the most salt-tolerant turfgrass species, can resist injury under 1% salt solution (Li et al., 2004). Zoysiagrass is now widely used in football pitches, home lawn, and ecological management (Teng et al., 2017). Studies conducted on this species so far have focused on the evaluation of abiotic stress tolerance among different cultivars, on reporting physiological mechanisms, and on the development of molecular markers (Guo et al., 2014; Xu et al., 2015). Thus, the molecular mechanism of salt tolerance in zoysiagrass remains unclear, mostly due to limited genetic resources. Functional studies of individual C_2_H_2_ zinc finger proteins will not only provide a better understanding of its detailed function in plant adaption to stress, but also provide insight into potential signaling processes occurring in plants under stress conditions. Therefore, the present study aimed to identify a C_2_H_2_ zinc finger protein in zoysiagrass, explore its role in salt stress tolerance and reveal its transcriptional regulation mechanism.

## Materials and Methods

### Plant Materials and Growth Conditions

*Zoysia japonica* cultivar “Companion” seeds were purchased from Hancock seed company (Dade City, FL, United States) and grown in a greenhouse at 28/25°C (day/night) under 400 μmol.m^-2^.s^-1^ photosynthetically active radiation (PAR). *Nicotiana benthamiana* seedlings were grown in a growth chamber set at 22°C with a photoperiod of 16 h. *Arabidopsis thaliana* ecotype “Columbia” (wild type, WT) plants were kept at 24/22°C (day/night) with 65% relative humidity and a scotophase of 8 h to yield transgenic lines. Transgenic lines were cultivated under the same growth conditions as the WT plants and T_3_-generation seeds were harvested for phenotype observation. The plants were manually irrigated with 1/2 strength Hoagland’s nutrient solution weekly (Hoagland and Arnon, 1950).

### Isolation of *ZjZFN1*

Total RNA was extracted from “Companion” leaves using Trizol (Invitrogen, Carlsbad, CA, United States) according to the manufacturer’s instructions. Using the rapid amplification of cDNA ends (RACE) method, 5′/3′ full-length sequences of *ZjZFN1* were isolated with a SMARTer RACE Kit (TaKaRa, Dalian, China) following the manufacturer’s instructions. Primers used for 5′/3′-RACE (**Table 1**) were designed based on a known cDNA sequence fragment (comp219112_c0) screened from our previous RNA-seq database (Xie et al., 2015). The amplified products were purified before the transfer into pMD18-T vectors (TaKaRa, Dalian, China). Positive clones were then sequenced at Rui-Biotech Company (Beijing, China). Based on the resulting sequences, the gene specific primers, ZjZFN1-F and ZjZFN1-R, for amplification of full-length *ZjZFN1* cDNA and genomic DNA sequences.

**Table 1:** Primers used for gene cloning, qRT-PCR detection, and plasmid construction.

Primer name	Primer sequence (5′–3′)
5′RACE	CAGATGGAGCAGCGGTGGACT
3′RACE	GGCAAGTCGTTCGGCTCCT
ZjZFN1-F	ATGTCGTCCGCCATGGAATT
ZjZFN1-R	TCACGCGGTCATGAGGAGGC
ZFN1-R1	GTAGGAGCCGAACGACTTGC
ZFN1-R2	GAGGCAGAGCGCGAGGTTCT
ZFN1-R3	CTTCTCCCTCTGGTGGTGCT
Promoter-F	TGATCTGATCCCATCGTCCCT
Promoter-R	TCCTTCTCCCTCTGGTGGTGCT
qZFN1-F	GCACCACCAGAGGGAGAAGGA
qZFN1-R	GGTAGGAGCCGAACGACTTGC
3302Y-ZFN1-F	cacgggggactcttgaccatggtaATGTCGTCCGCCATGGAATT
3302Y-ZFN1-R	ggtacacgcgtactagtcagatcCGCGGTCATGAGGAGGCGGG
BD-ZFN1-F	tggccatggaggccgaattcccgATGTCGTCCGCCATGGAATT
BD-ZFN1-R	tgcggccgctgcaggtcgacgCGCGGTCATGAGGAGGCGGG
1391Z-ZFN1-F	aagcctagggaggagtccacTGATCTGATCCCATCGTCCCT
1391Z-ZFN1-R	tttaccctcagatctaccatCGCCGTTGGCTCGATCGGCGA
AtUBQ-F	AGTCCACCCTTCATCTTGTTCTC
AtUBQ-R	GTCAGCCAAAGTTCTTCCATCT
AtMn-SOD-F	CGCATGATCCTTTGGCTTCG
AtMn-SOD-R	TCCTGGTTGGCTGTGGTTTC
AtPOD-F	CCAAACTCTTCGTGGACTATGC
AtPOD-R	AACTCTTGGTCGCTCTGGAT
AtAPX1-F	CTCTGGGACGATGCCACAAG
AtAPX1-R	CTCGACCAAAGGACGGAAAA
AtNHX1-F	AGCCTTCAGGGAACCACAAT
AtNHX1-R	CTCCAAAGACGGGTCGCATG
AtP5CS-F	GGGACAAGTTGTGGATGGAGAC
AtP5CS-R	TGGTACAAACCTCAAGGAACAC
AtLEA-F	GATTGACCCGGCTGAGCTACGA
AtLEA-R	AGATGGGATTCACCACAAAAG

### Isolation of the *ZjZFN1* Promoter

Genomic DNA was extracted from “Companion” leaves using the cetyl trimethylammonium bromide (CTAB) method and used as template for genome walking (Genome Walking Kit, TaKaRa, Dalian, China) according to the instruction. The gene specific primers ZFN-R1, ZFN-R2, and ZFN-R3 were used in the genome walking. The resulting PCR products were sequenced using the ZFN-R3 primer. Finally, promoter specific primers, namely Promoter-F and Promoter-R, were used to amplify the upstream sequence of *ZjZFN1*.

### Bioinformatics Analysis

The ZjZFN1 amino acid sequence was deduced from the corresponding cDNA sequence using DNAMAN software (v. 7.0). Its theoretical isoelectric points (pI) and molecular weight (MW) were calculated using the Compute pI/MW tool^1^. Subcellular localization character was predicted using ProtComp 9.0^2^. Promoter *cis*-regulatory elements analysis was carried out via PLACE database^3^. Potential signal peptide cleavage sites were examined with SignalP 4.1 Server^4^. The basic local alignment search tool (BLAST) at the National Center for Biotechnology Information (NCBI) database was used to identify homologs. Clustal W^5^ was employed to perform protein alignment. A phylogenetic tree was built in MEGA version 5.0 based on the neighbor-joining method.

### Quantitative Real-Time PCR

The expression patterns of *ZjZFN1* in the roots, stems, and leaves at different developmental stages (young, fast-growing, and mature) of *Z. japonica* were explored using qRT-PCR. Furthermore, *ZjZFN1* expression profiles were investigated in 3-month-old *Z. japonica* after 24 h’ under 8°C, 150 mM NaCl, 25% PEG6000, 10 μM ABA, 5 μM SA, or 10 μM MeJA. The *ZjZFN1* specific primers qZFN-F and qZFN-R were used for qRT-PCR (**Table 1**). Reactions were performed in 96-well blocks with a CFX Connect RT-PCR system (BIO-RAD, Hercules, CA, United States) using SYBR Premix (TaKaRa, Dalian, China) in a total volume of 25 μL. A two-step qRT-PCR program was adopted and set as follows: initial denaturation at 95°C for 30 s, followed by 40 cycles of 95°C for 5 s and 60°C for 30 s. The *Z. japonica actin* gene (GenBank accession GU290546) was selected as the internal reference (**Table 1**) and the relative gene expression levels were calculated using the comparative ΔΔCt method (Livak and Schmittgen, 2001). All data are presented as means [with corresponding standard deviations (SDs)] of at least three independent biological replicates, each including three technical replicates. To obtain sufficient samples for RNA extraction, three independent plants were pooled per biological replicate.

### Binary Vector Construction

The yellow fusion protein (YFP) construct *35S::ZjZFN1:YFP* and pGBKT7-ZjZFN1 were produced by transferring the complete *ZjZFN1* coding sequence (CDS) into 3302Y (Jia et al., 2016) and pGBKT7 vectors, respectively. Firstly, 3302Y and pGBKT7 vector were digested by *Bgl*II or *Bam*HI (TaKaRa, Dalian, China), respectively, and purified using a E.Z.N.A Cycle-pure Kit (Omega Bio-Tek, Norcross, GA, United States). Then primers 3302Y-ZFN1-F/R and BD-ZFN1-F/R were used to amplify the *ZjZFN1* CDS (**Table 1**). Amplicons were then purified and infused into the linearized 3302Y and pGBKT7 plasmids using an In-fusion HD Cloning Kit (TaKaRa, Dalian, China).

The GUS fusion construct *ZjZFN1_pro_::GUS* contained a 1406-bp *ZjZFN1* promoter region that was amplified from the plasmid containing the target sequence using primers 1391-ZFN1-F and 1391-ZFN1-R (**Table 1**). After digesting the pCAMBIA1391Z vector with *Nco*I (TaKaRa, Dalian, China), purified PCR product of *ZjZFN1* promoter was infused into the digested vector using the In-fusion HD Cloning Kit (TaKaRa, Dalian, China) to produce *ZjZFN1_pro_::GUS*.

### Subcellular Localization and Transcriptional Activity Assay of ZjZFN1

*Agrobacterium tumefaciens* EHA105 transformed with the *35S::ZjZFN1:YFP* fusion construct were used to transform *N. benthamiana* to reveal the subcellular localization of ZjZFN1 using the transient overexpression method (Sparkes et al., 2006). After darkness induction for 48 h, the cells from the lower epidermis of *N. benthamiana* leaf cells were monitored and photographed under a SP-5 laser confocal scanning microscope (Leica, Mannheim, Germany). 1 μg/mL DAPI (Sigma-Aldrich, Munich, Germany) was used to show the nuclear.

The pGBKT7-ZjZFN1 construct was transformed into *Saccharomyces cerevisiae* Y2HGold competent cells using the LiAc method (Liu et al., 2016) to investigate its transcriptional activation ability. Firstly, transformed Y2HGold cells (pGBKT7 was use as control) were grown on synthetic defined (SD) medium without tryptophan (SD/-Trp). After colony-PCR verification, the positive clones were grown on SD medium without tryptophan, histidine and adenine (SD/-Trp-His-Ade) at 30°C for about 48 h. Yeast growth phenotypes were then photographed with an EOS 60D digital camera (Canon, Tokyo, Japan).

### Generation of Transgenic Plants

Using floral dip method (Clough and Bent, 1998), *A. tumefaciens* GV3101 transformed with construct plasmids was used to infect Arabidopsis plants to generate transgenic plants expressing *ZjZFN1* or *ZjZFN1_pro_::GUS*. Transgenic Arabidopsis seeds were screened using 60 mg L^-1^ glufosinate, or 20 μg/mL hygromycin. Positive transgenic plants were verified by reverse transcription PCR and genomic PCR. Representative T_3_ transgenic lines exhibiting 100% resistance to glufosinate were harvested for further phenotype observation or GUS staining assays.

### GUS Staining

Using a GUS Kit (O’BioLab, Beijing, China), and according to the instructions provided by the manufacture, Arabidopsis seedlings were GUS stained. After removing chlorophyll with 70% ethanol, seedlings were then photographed under the M205FA stereomicroscope (Leica, Mannheim, Germany).

### Salt Tolerance in Arabidopsis Transgenic Lines

Because lines ZFN-2 and ZFN-17 presented the highest *ZjZFN1* transcript levels among the 36 T_3_ transgenic lines, they were selected as the representative lines for phenotype observation. For germination assessment, seeds of ZFN-2, ZNF-17, and WT were sterilized with 70% ethanol and 1% sodium hypochlorite and then sowed on Murashige and Skoog (MS) medium with or without 100 mM NaCl, respectively. After 4 and 8 days, phenotype were photographed with an EOS 60D digital camera (Canon, Tokyo, Japan). For determination salt tolerance, 3-week-old seedlings were transplanted to nutrition medium containing peat, vermiculite and pearlite (1:1:1 in volume) and moved to growth chambers. Here, seedlings were subject to incremental increases of 50 mM NaCl from the first day to the third day and then kept under 150 mM NaCl for 18 days. Leaf samples were then harvested for malondialdehyde (MDA), proline and gene expression examination at the 21st day.

For MDA and proline quantification, the leaf samples were treated as described in our previous report (Teng et al., 2017). Genes coding for stress response proteins, namely superoxide dismutase (*AtMn-SOD*, AT3G56350), peroxidase (*AtPOD*, AT3G49120), ascorbate peroxidase1 (*AtAPX1*, AT1G07890), sodium/hydrogen exchanger 1 (*AtNHX1*, AT5G27150), pyrroline-5-carboxylate synthase (*AtP5CS*, AT3G55610), and late embryogenesis abundant (*AtLEA*, AT1G02820) were selected to monitor salt stress-response transcriptional mechanisms using qRT-PCR (**Table 1**). The Arabidopsis *AtUBQ10* gene (NM_116771) gene was adopted as internal reference.

### Transcriptomic Analysis

Total RNA was isolated from 4-week-old WT (control) and *ZjZFN1*-overexpressing Arabidopsis seedlings (ZFN-17) cultivated under normal conditions using Trizol (Invitrogen, Carlsbad, CA, United States). Six independent RNA samples (three replicates from control and three replicates from ZNF-17), each comprising three Arabidopsis plants were obtained. Nanodrop ND-1000 spectrophotometer (NanoDrop Technologies, Rockland, DE, United States) was used for RNA quality determination. Agilent 2100 spectrophotometer (Agilent Technologies, Santa Clara, CA, United States) was used to evaluate RNA purity, concentration and integrity. Sequencing libraries and sequencing analysis were performed by Biomarker Technologies (Beijing, China) on the Illumina 4000 platform (San Diego, CA, United States) to generate 125/150 bp paired-end reads. Raw sequence reads were deposited into the NCBI Short Read Archive (SRA) repository under the accession number SRP140821. The reads containing adaptor and poly-*N*, as well as the low quality reads, were excluded and the Q20, Q30, and GC content of clean data reads were evaluated. High-quality clean reads were then mapped to the Arabidopsis reference genome (TAIR 10^6^) using TopHat2 (Kim et al., 2013). Gene expression levels were calculated base on the fragments per kilobase of transcript per million fragments mapped (FPKM) method (Mortazavi et al., 2008).

Differentially expressed genes between the WT and the ZFN-17 line (each with three independent libraries) were screened using DESeq R package (1.10.1) based on adjusted false discovery rate (FDR). *P*-value ≤0.05 and fold change (FC) ≥2. For GO and KEGG enrichment analyze, all DEGs were mapped to GO terms in the GO database and KEGG database using the Goseq R package (Young et al., 2010) and the KOBAS software (Mao et al., 2005), respectively.

### Statistical Analysis

Data were analyzed by two-tailed Student’s *t* test or one-way ANOVA using SPSS version 18.0 (IBM, Chicago, IL, United States). ^∗^*P* < 0.05 and ^∗∗^*P* < 0.01 were believed statistically significant. All data were presented as the means with ± SD (*n* = 3).

## Results

### Isolation and Bioinformatics Analysis of *ZjZFN1* and Its Promoter

**FIGURE 1:**
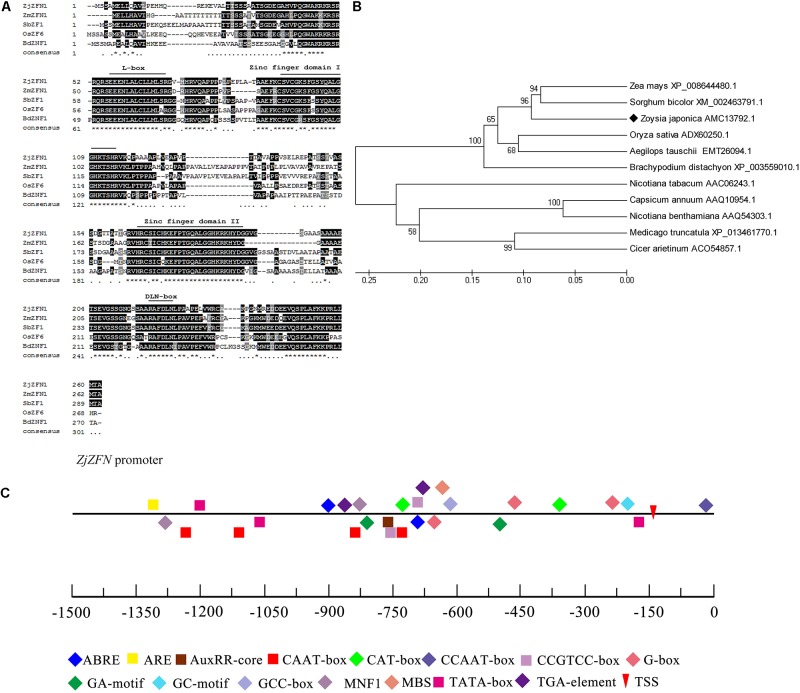
Amino acid sequence and phylogenetic analysis of ZjZFN1. **(A)** The alignment of C_2_H_2_-zinc finger proteins. The position of the domain of the first and second zinc fingers, L-box and DLN-box were labeled. **(B)** Phylogenetic analysis of ZjZFN1 protein orthologs. **(C)** *Cis*-elements analysis of *ZjZFN1* promoter sequence.

The *ZjZFN1* cDNA sequence was deposited in the NCBI database with the accession number of KT596064.1. The open reading frame (ORF) of *ZjZFN1* was 789 bp in length, which corresponded to 262 amino acids (**Figure 1**). The ZjZFN1 protein contained two typical zinc finger structures and belonged to the C_2_H_2_ superfamily (**Figure 1A**). Its theoretical pI was 8.76. Its MW was 27.52 KD and no potential signal peptide was found. Phylogenetic analysis showed that ZjZFN1 was most closely related to the ZFN proteins from *Zea mays* or *Sorghum bicolor* (**Figure 1B**).

The 1406-bp fragment upstream the ATG start codon, containing CAAT-box and TATA-box elements, was investigated as a potential promoter region (**Figure 1C**). Two ABA-responsive motifs (ABRE) and one auxin-responsive motif (AuxRR-core) were also identified. In addition, there were one *cis*-acting regulatory element related to meristem expression (CAT-box) and two involved in anaerobic induction (ARE).

### Expression Patterns of *ZjZFN1*

**FIGURE 2:**
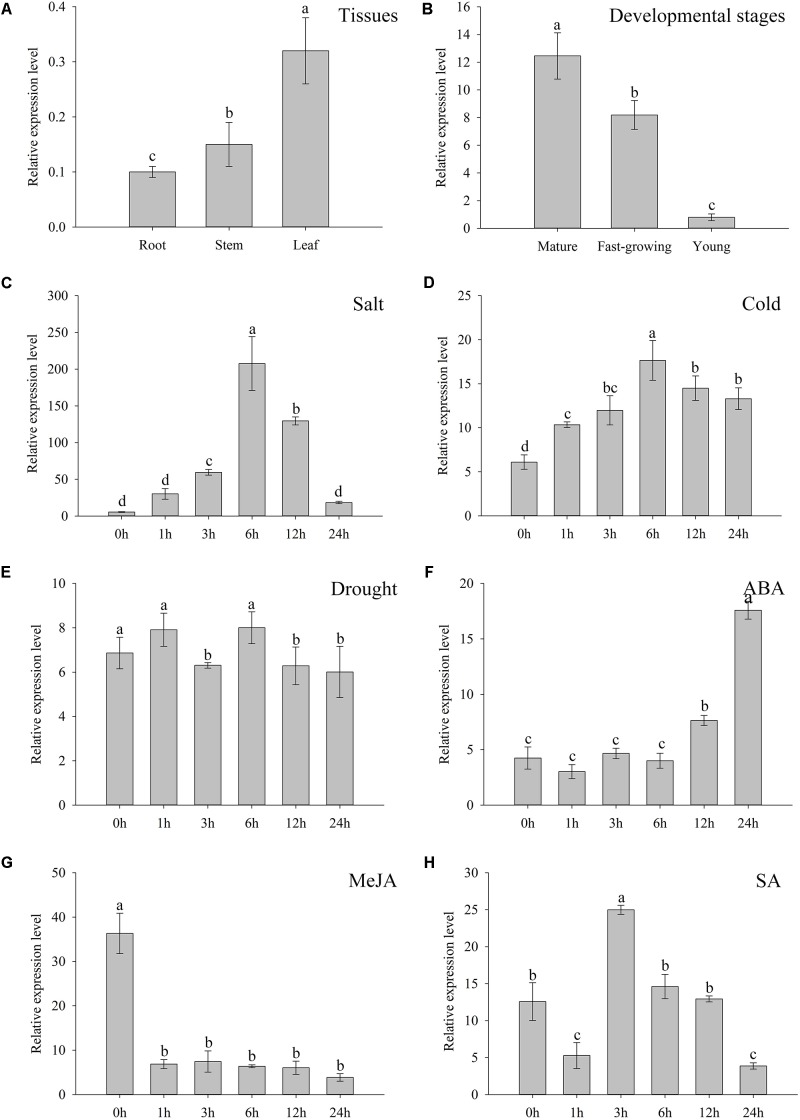
Expression characters of *ZjZFN1*. **(A)** *ZjZFN1* expression levels in root, stem, and leaf of *Z. japonica*. **(B)** Expression levels of *ZjZFN1* in *Z. japonica* leaves at different developmental stages. **(C–F)** *ZjZFN1* expression levels in *Z. japonica* exposed to salt **(C)**, cold **(D)**, drought **(E)**, 10 μM ABA **(F)**, 10 μM MeJA **(G)**, and 0.5 mM SA **(H)**. Mean ± SDs (*n* = 3). Different letters indicate significant differences at 5% level of probability.

The qRT-PCR employed to interpret the expression patterns of *ZjZFN1* showed that, although *ZjZFN1* expressed in all investigated tissues including leaf, stem, and root, most transcripts were observed in leaves (**Figure 2A**). The expression level of *ZjZFN1* was higher in mature leaves than in fast-growing and young leaves (**Figure 2B**). The transcription of *ZjZFN1* could be induced by cold and salt treatments, as the highest expression levels were observed at 6 h after NaCl or cold treatments, respectively (**Figures 2C,D**). However, we found that *ZjZFN1* was not suspected to drought treatment in this study (**Figure 2E**). In addition, *ZjZFN1* expression was considerably elevated by exogenous ABA, with highest transcript abundance at 24 h after 10 μM application (15.68-fold higher than that at 0 h) (**Figure 2F**). On the contrary, *ZjZFN1* expression was prone to suppression by 10 μM MeJA during 24 h after its application (**Figure 2G**). Although 0.5 mM SA suppressed the expression of *ZjZFN1* 1 h after application, it then stimulated the expression of *ZjZFN1*, which was highest 3 h after MeJA application (**Figure 2H**).

### ZjZFN1 Was Localized to Nucleus and Had Transcriptional Activity

**FIGURE 3:**
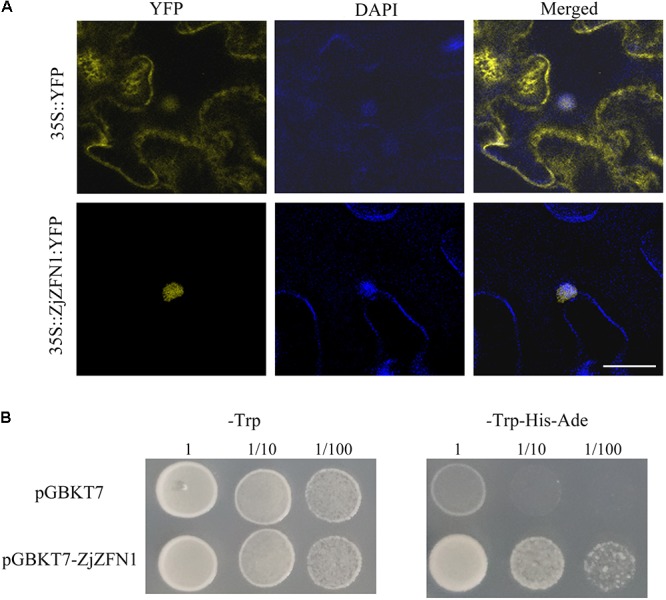
Subcellular localization and transcriptional activation ability assay of ZjZFN1. **(A)** Subcellular localization of ZjZFN1 under UV radiation in *N. benthamiana* leaf epidermis. Fluorescence of *35S::ZjZFN1:YFP* was dominantly distributed in the nucleus. **(B)** Transcriptional activation ability assay using Y2HGold yeast system.

Bioinformatics analysis showed that ZjZFN1 contained a nuclear localization signal. To confirm the exact subcellular localization of ZjZFN1, we performed transient overexpression of *35S::ZjZFN1:YFP* in *N. benthamiana* leaf cells. Laser confocal microscopic observations revealed strong YFP signal in the whole cells of the control while the YFP signal was only found in nucleus in *35S::ZjZFN1:YFP* overexpressing cells (**Figure 3A**). It proved that ZjZFN1 was localized to the nucleus.

The Y2HGold yeast cells transformed with pGBKT7-ZjZFN1 and pGBKT7 empty vectors could grow normally on SD/-Trp medium (**Figure 3B**) suggesting that the target plasmids were successfully transformed in the host cells. Further analysis showed that the yeast cells transformed with the pGBKT7-ZjZFN1 vector could grow on SD/-Trp-His-Ade medium whereas control yeast cells (**Figure 3B**). Thus ZjZFN1 had transcriptional activation ability.

### Abundant *ZjZFN1* Expression in Leaf

**FIGURE 4:**
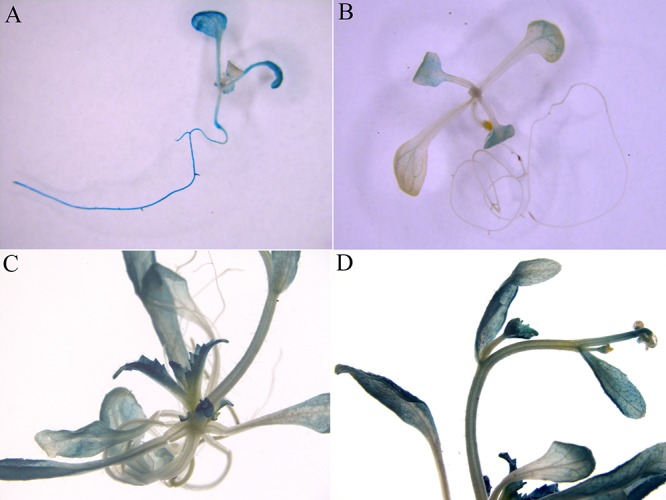
β-Glucuronidase (GUS) staining in transgenic Arabidopsis seedlings. **(A)** *35S::GUS* (control), **(B)** 2-week-old *ZjZFN1_Pro_::GUS*-overexpressing Arabidopsis seedlings, **(C,D)** Mature *ZjZFN1_Pro_::GUS*-overexpressing Arabidopsis seedlings.

The native promoter of *ZjZFN1* was infused with *GUS* to generate the *ZjZFN1pro::GUS* construct, and transgenic Arabidopsis lines were generated to examine promoter activity. Twenty-two independent transgenic lines were examined for GUS staining. Histochemical analysis showed obvious GUS signal in the seedlings transformed with the *35S::GUS* construct (control) (**Figure 4A**). However, strong GUS activity was only detected in the leaves and petioles of the *ZjZFN1pro::GUS* transgenic seedlings (**Figures 4B–D**). These results proposed that *ZjZFN1* was mostly expressed in leaf and petioles.

### *ZjZFN1* Positively Regulated Seed Germination Under Salt Stress

**FIGURE 5:**
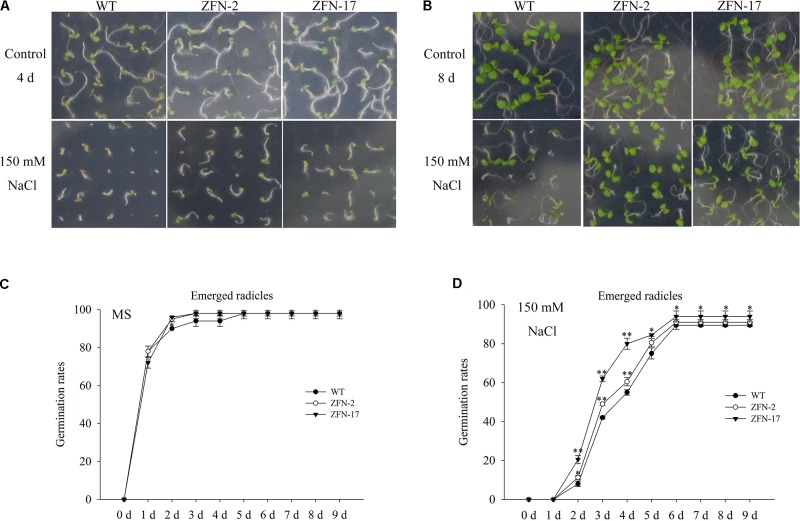
Germination assay of *ZjZFN1*-overexpressing lines in 150 mM NaCl. Growth of the transgenic and WT plants in the presence of 150 mM NaCl for 4 days **(A)** and 8 days **(B)**, respectively. Rates of emerged radicles of control and the transgenic plants in MS medium **(C)** and MS medium containing 150 mM NaCl **(D)**.

Arabidopsis plants overexpressing *ZjZFN1* were generated to characterize the function of *ZjZFN1* in response to salt stress. No significant difference in germination determination was observed between WT and transgenic lines in the absence of NaCl (**Figures 5A–C**). However, the *ZjZFN1*-overexpressing lines showed enhanced salt tolerance in relation to that of WT in MS medium containing 150 mM NaCl before day 6. In detail, on the fourth day after being sowed in the presence of 150 mM NaCl, approximately 60% of WT seeds germinated with emerged radicles, while nearly 75% transgenic seeds germinated with emerged radicles (**Figure 5D**). Obvious differences were also observed in terms of the percentage of green cotyledons at the eighth day after sowing (**Figure 5B**).

### Overexpression of *ZjZFN1* Enhanced Plant Tolerance to Salt Stress

**FIGURE 6:**
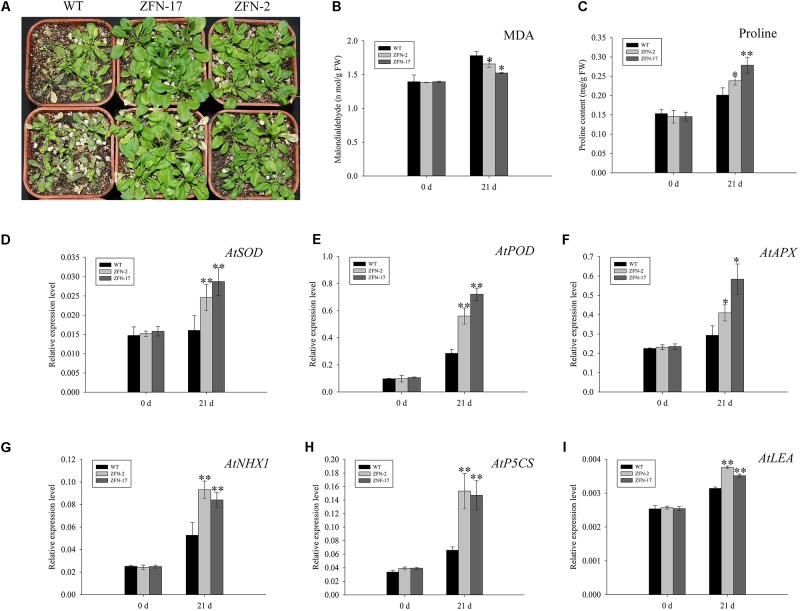
Phenotype observation and indexes determination. **(A)** Phenotype of transgenic lines and control under 150 mM NaCl treatment for 21 days. **(B)** Comparison of MDA. **(C)** Proline contents. **(D)** *AtSOD* expression. **(E)** *AtPOD* expression. **(F)** *AtAPX* expression. **(G)** *AtNHX1* expression. **(H)** *AtP5CS* expression. **(I)** *AtLEA* expression. ^∗^ and ^∗∗^, respectively, represent significant differences from the control at values of *P* < 0.05 and *P* < 0.01 as determined by Student’s *t*-test.

To further examine the function of *ZjZFN1*, 3-week-old *ZjZFN1*-overexpressing plants were subjected to 150 mM NaCl for 21 days. All surveyed plants showed delayed growth and leaves were dehydrated, although ZFN-2 and ZFN-17 plants showed less damage compared to that observed in WT plants (**Figure 6A**). Accordingly, no significant difference in MDA or proline content was detected between *ZjZFN1*-overexpressing and WT plants under normal conditions. Nevertheless, MDA contents in WT increased to 1.78 nmol g^-1^, which was 7.5 and 16.9% higher than that in ZFN-2 and ZFN-17, respectively, at the end of the experiment (**Figure 6B**). In addition, under salt stress, ZFN-2 and ZFN-17 showed 15.6 and 27.7% increased proline content in relation to that of WT, respectively (**Figure 6C**).

To interpret the underlying transcriptional mechanism, a qRT-PCR was performed to evaluate gene expression differences between *ZjZFN1*-overexpressing and WT plants. Six salinity responsive genes involved in antioxidation (*AtSOD, AtPOD*, and *AtAPX*), ion transport (*AtNHX1*), and osmotic regulation (*AtP5CS* and *AtLEA*) were selected to monitor transcriptional variation. No obvious difference in gene expression was found between WT and transgenic plants under normal growth conditions. However, under salinity stress, the transcription of all genes was up-regulated, particularly in *ZjZFN1*-overexpressing plants (**Figures 6D–I**).

### Global Expression Analysis of Abiotic Stress-Related Genes in *ZjZFN1*-Overexpressing Plants

**FIGURE 7:**
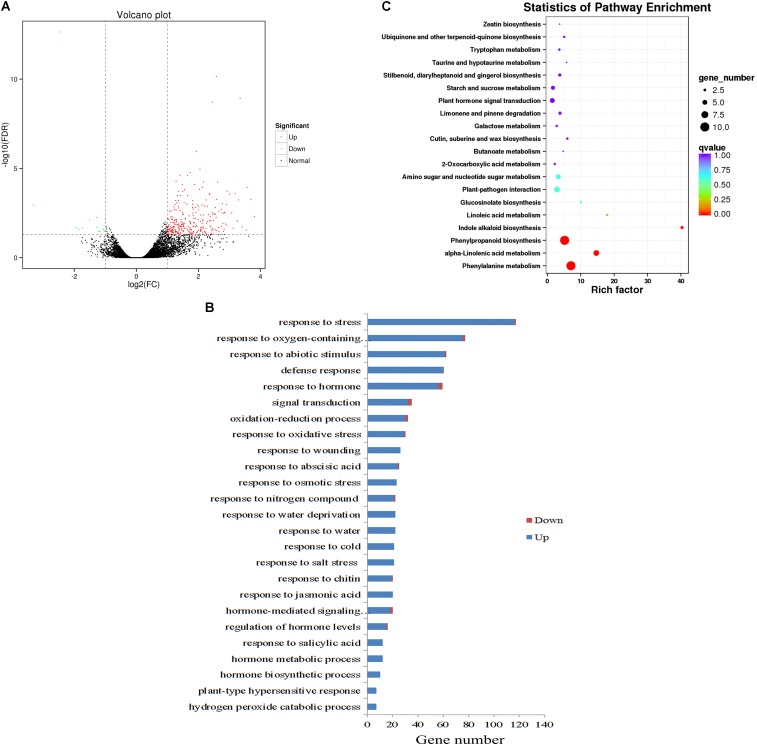
Global transcriptome analysis of WT and *ZjZFN1*-overexpressing Arabidopsis plants. **(A)** Volcano plot of the DEGs. **(B)** Gene ontology (GO) classification for abiotic stress-related DEGs. Only the biological processes were collected for GO term analysis. **(C)** Statistic of KEGG pathway enrichment.

To provide a comprehensive landscape of the transcriptional regulation network of *ZjZFN1* and gain insight into the performance of *ZjZFN1*-overexpressing plants, we carried out an RNA-seq assessment to screen DEGs between WT and transgenic plants (ZFN-17). Among the 289 DEGs identified, 274 were up-regulated and 15 were down-regulated in ZFN-17 (**Figure 7A** and **Supplementary Table S1**). The DEGs were classified into 51 groups based on their allocated GO terms (**Supplementary Figure S1**). Further classification of the DEGs within the “biological process” group revealed a large number of abiotic stress responsive DEGs including “response to stress,” “response to abiotic stimulus,” and “response to salt stress” (**Figure 7B**). All the 21 DEGs classified within the “response to salt stress” term were up-regulated, and these included genes *SOS2, WRK33, MYB15*, and *peroxidase (isoforms 22 and 23)*, among other genes (**Table 2**).

**Table 2:** DEGs classified into the GO term of “response to salt stress.”

AGI	Log_2_ FC	*P*-value	Annotation
AT1G03220	1.501511849	0.01388	Aspartyl protease-like protein
AT2G38380	2.589680215	0.000544	Peroxidase 22
AT3G08720	1.439202055	0.010174	Serine/threonine protein kinase 2
AT2G38470	1.488299735	0.002867	Putative WRKY transcription factor 33
AT5G26340	1.379047294	0.022802	Sugar transport protein 13
AT3G48360	1.404194641	2.43E-05	TAC1-mediated telomerase activation pathway protein BT2
AT3G57530	1.120060074	0.005128	Calcium-dependent protein kinase 32
AT4G12480	2.158267439	5.76E-05	Putative lipid transfer protein
AT3G23250	1.963947163	0.001117	MYB domain protein 15
AT5G49480	1.462536757	0.028422	Ca^2+^-binding protein 1
AT4G11650	2.097296486	0.029137	Osmotin-like protein OSM34
AT2G33380	1.933768292	0.015409	Caleosin 3
AT3G61890	1.553503187	0.022474	Homeobox-leucine zipper protein ATHB-12
AT2G38390	3.806701278	0.004953	Peroxidase 23
AT4G19810	1.750323814	0.011371	Class V chitinase
AT2G41010	1.122992269	0.030247	Calmodulin binding protein 25
AT2G15390	1.323281753	0.013962	Probable fucosyltransferase 4
AT4G34710	1.141893988	0.034177	Arginine decarboxylase 2
AT4G23600	1.188045432	0.021944	Cystine lyase CORI3
AT3G25780	1.512956793	0.034314	Allene oxide cyclase 3
AT5G59820	1.459308538	0.014216	Zinc finger protein ZAT12

All DEGs were analyzed regarding the pathways underlying the regulation of *ZjZFN1*. The 36 enriched KEGG pathways included 33 metabolic pathways (**Supplementary Figure S1**). Among these, the “phenylalanine metabolism” and “phenylpropanoid biosynthesis” pathways, both containing 10 annotated DEGs, were the most abundant pathways. Statistics of pathway enrichment revealed that “phenylalanine metabolism,” “alpha-linolenic acid metabolism,” “phenylpropanoid biosynthesis,” and “indole alkaloid biosynthesis” pathways were significantly enriched (Corrected *P* < 0.05) (**Figure 7C**).

## Discussion

Genetic studies on halophytic species lag far behind those in other species and their potential remain unexplored (Orsini et al., 2010). Zinc finger proteins, a large gene family in plants, plays a key role in environmental adaption. Here, we isolated a novel zinc finger protein, *ZjZFN1*, from *Z. japonica* aiming to better understand its function and underlying transcriptional regulation mechanism. Homology analysis showed that ZjZFN1 belonged to the C_2_H_2_ zinc finger protein family and contained two typical zinc finger structures, suggesting its potential for high DNA binding affinity (Kielbowicz-Matuk, 2012). The localization of ZjZFN1 to the nucleus was consistent with that of ZAT6 (Shi et al., 2014) and ZAT18 (Yin et al., 2017) in Arabidopsis, OsMSR15 in *Oryza sativa* (Zhang X. et al., 2016), and VvZFP11 in *Vitis vinifera* (Yu et al., 2016). The yeast assay provided evidence that ZjZFN1 has transcriptional activation ability, which was consistent with the results obtained for VvZFP11 and OsMSR15. As extensively reported, the nuclear localization attribute together with transcriptional activation ability suggest that ZjZFN1 might function as a typical plant TF.

Although *ZjZFN1* was widely expressed in different tissues of *Z. japonica*, the highest expression levels were found in leaves. Additionally, *ZjZFN1* transcript abundance was highest in mature leaves. In agreement, GUS staining results corroborated those of the qRT-PCR, suggesting that *ZjZFN1* might be related with leaf development. The motifs exhibited in the *ZjZFN1* promoter suggested that its expression might be regulated by different abiotic and hormone factors. In the present study, the transcription of *ZjZFN1* was induced by cold and salt treatments but not by drought stress, which is consistent with that found for *ZAT6, ZAT10*, and *ZAT18* (Yin et al., 2017), except for drought stress. Hormone induction experiments revealed that *ZjZFN1* expression changed in response to SA, which is consistent with the results obtained for *AtZAT6* (Shi et al., 2014) and VvZFP11 (Yu et al., 2016). The expression level of *ZjZFN1* was also up-regulated by exogenous ABA, in agreement with the attributes of *GmZFP3* (Zhang D.Y. et al., 2016), *StZFP1* (Tian et al., 2010), and *TaDi19A* (Li et al., 2010). These results indicate that *ZjZFN1* might play several roles in plants’ response to environmental stresses and might be an important factor for signal transduction.

C_2_H_2_ zinc finger proteins have key roles in the response to salinity stress in different plant species. In the present study, the germination and growth of *ZjZFN1*-overexpressing lines was obviously improved under salinity stress compared with that of WT plants. MDA is regarded as a typical indicator of salinity stress (Shi et al., 2013b). Additionally, increase in proline content is a primary metabolic defense for protecting plants from abiotic stresses (Zhang et al., 2011). Consistent with phenotype observations, transgenic lines showed lower MDA but higher proline content than WT plants under salt stress, supporting the hypothesis that *ZjZFN1* overexpression of enhances plant tolerance to salinity stress.

In plant responses to environmental stresses, transcriptional regulation plays a dominant role (Teng et al., 2017). Under salt stress, plants employ reactive oxygen species (ROS) scavenging enzymes, such as SOD, POD, and APX, to eliminate ROS accumulation. The improved transcript abundance of *SOD, POD*, and *APX* reflected an enhanced ROS-scavenging ability in *ZjZFN1*-overexpressing lines, which was consistent with that reported for *ZFP179* (Sun et al., 2010). As an important ion transporter, the overexpression of *NHX1* improves plant resistance to salinity stress (Shi et al., 2003). P5CS activates proline biosynthesis to protect protein integrity and enhance the activities of different enzymes under osmotic stress (Szabados and Savoure, 2010). LEA contributes to plant responses to abiotic stresses (Olvera-Carrillo et al., 2010). In the present study, the overexpression of *ZjZFN1* induced the transcription of all selected genes under salinity stress in relation to shown by control (WT) plants, suggesting that *ZjZFN1* may regulate salt tolerance via multiple pathways, in agreement with phenotype observations.

Genes encoding stress-activated C_2_H_2_ TFs also fine-tune the transcription of stress-responsive genes, leading to parallel changes in plant adaptation to various stresses (Kielbowicz-Matuk, 2012; Yin et al., 2017). Transcriptome analysis is a powerful tool for the identification of novel C_2_H_2_-manipulating genes. Among the 289 screened DEGs, 274 (94.81%) were up-regulated and 15 (5.19%) were down-regulated by *ZjZFN1*, corroborating that *ZjZFN1* may function as a transcriptional activator. Specifically, some of the 21 DEGs enriched in the “response to salt stress” term, corresponded to genes *SOS2, peroxidase (isoforms 22 and 23), WRKY33*, and *MYB15*, are directly involved in plant salt tolerance. Increasing evidence has shown that phenylalanine ammonia-lyase (PAL) metabolism pathway actively participates in plant environmental stresses (Chen et al., 2008). Phenylpropanoid is an important enzyme involved in plant cell defense and response to salinity stress (Gao et al., 2008). Alpha-linolenic acid metabolism is one of the major fatty acids in leaf membrane lipids, and it has been reported to contribute to the osmotic tolerance of rice (Lenka et al., 2011). Pathway enrichment analysis demonstrated that the three pathways above were over-represented in *ZjZFN1*-overexpressing plants, indicating that *ZjZFN1* could modulate stress responsive pathways, which in turn contribute to the improved adaptive capacity of transgenic plants.

## Conclusion

In summary, we isolated *ZjZFN1* and interpreted its functions in salinity stress responses. The overexpression of *ZjZFN1* improved stress responses in seed germination and enhanced salt tolerance of transgenic lines. The *ZjZFN1* transgene decreased leaf MDA content and improved proline content, and improved transcriptional activities of several salt-stress-related genes under salinity stress conditions. The RNA-seq results indicated that *ZjZFN1* could function as a transcriptional activator, actively participating in the improvement of stress responsive genes’ expression and regulating stress-responsive pathways, including phenylalanine metabolism, α-linolenic acid metabolism, and phenylpropanoid biosynthesis pathways. Overall, our results suggest that *ZjZFN1* might be a valuable gene in *Z. japonica* breeding projects.

## Author Contributions

KT and JW conceived the study and designed the experiments. KT and PT performed the experiments. KT and WG analyzed the data with suggestions by YY, XF, and JW. KT wrote the manuscript.

## Conflict of Interest Statement

The authors declare that the research was conducted in the absence of any commercial or financial relationships that could be construed as a potential conflict of interest.

## Footnotes

1 http://web.expasy.org/compute_pi/

2 http://www.softberry.com

3 http://sogo.dna.affrc.go.jp/cgi-bin/sogo.cgi?lang=en&pj=640&action=page&page=newplace

4 http://www.cbs.dtu.dk/services/SignalP/

5 https://www.genome.jp/tools-bin/clustalw

6 ftp://ftp.ensemblgenomes.org/pub/plants/release-25/fasta/arabidopsis_thaliana/

## Supplementary Material

FIGURE S1 Gene ontology and KEGG annotation of the identified DEGs. **(A)** GO classification. **(B)** KEGG annotation.

TABLE S1 List of differently expressed genes between ZjZFN1-overexpressing line (ZFN-17) and WT screened by RNA-sequencing.

## Abbreviations

ABA: abscisic acid
DAPI: 4′,6-diamidino-2-phenylindole
DEGs: differently expressed genes
GO: Gene ontology
GUS: β-glucuronidase
KEGG: Kyoto Encyclopedia of Genes and Genomes
MeJA: methyl jasmonate
qRT-PCR: quantitative real-time PCR
RNA-seq: RNA sequencing
SA: salicylic acid
TFs: transcript factors
