# Supplementary figures and images for: Heterologous Expression of a Novel Zoysia japonica C2H2 Zinc Finger Gene, ZjZFN1, Improved Salt Tolerance in Arabidopsis
Source: Front Plant Sci. 2018 Aug 14;9:1159. doi: 10.3389/fpls.2018.01159 (PMC6102363; doi:10.3389/fpls.2018.01159)

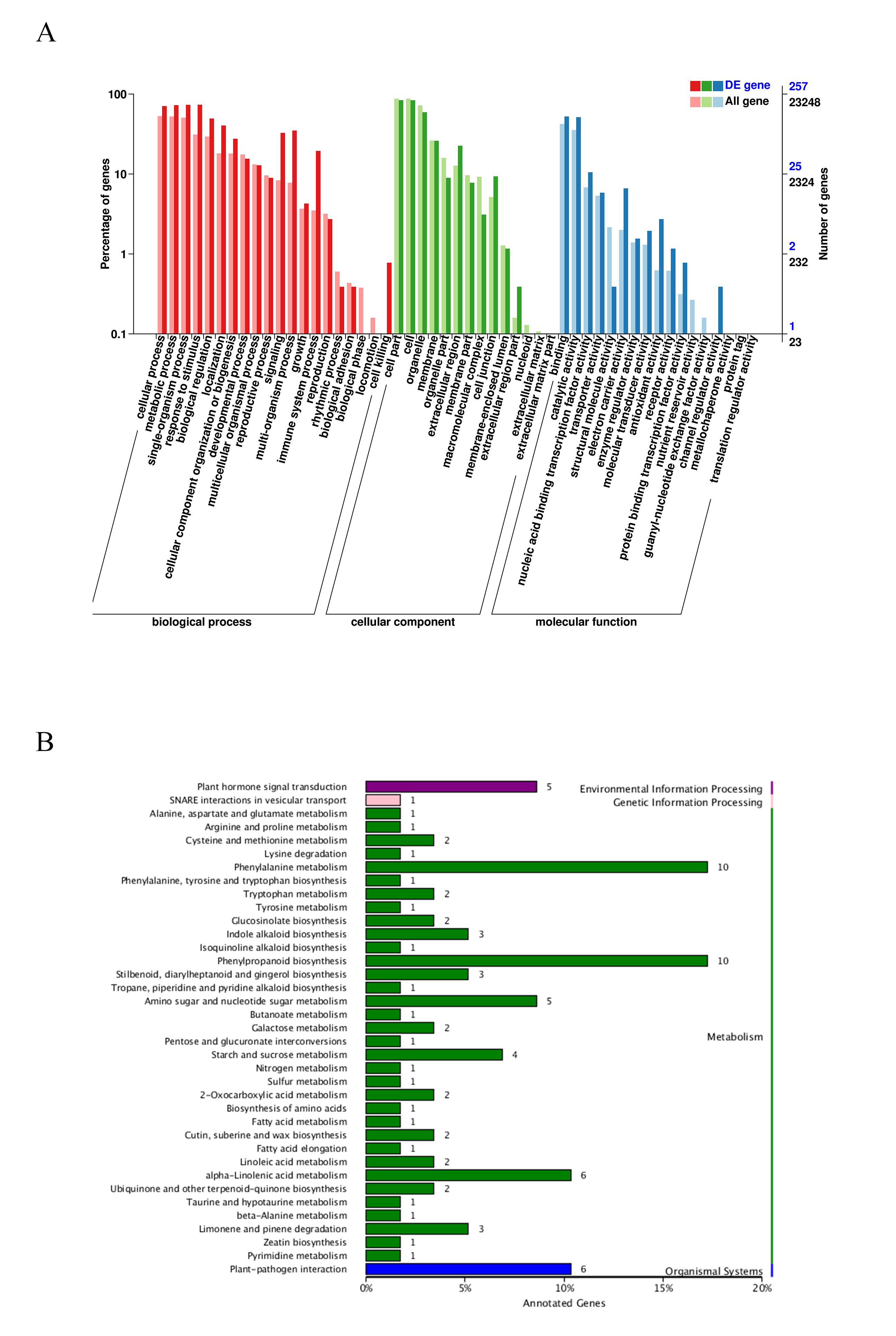

Supplement: FIGURE S1 — Gene ontology and KEGG annotation of the identified DEGs. (A) GO classification. (B) KEGG annotation. [file Image_1.JPEG]
